# Comparing the Convergent and Concurrent Validity of the Dynamic Gait Index with the Berg Balance Scale in People with Multiple Sclerosis

**DOI:** 10.3390/healthcare7010027

**Published:** 2019-02-15

**Authors:** Tapan Mehta, Hui-Ju Young, Byron Lai, Fuchenchu Wang, Yumi Kim, Mohan Thirumalai, Tracy Tracy, Robert W. Motl, James H. Rimmer

**Affiliations:** 1 Department of Health Services Administration, School of Health Professions, University of Alabama at Birmingham, Birmingham, AL 35294, USA; mohanraj@uab.edu; 2 UAB/Lakeshore Research Collaborative; School of Health Professions, University of Alabama at Birmingham, Birmingham, AL 35294, USA; hjyoung@uab.edu (H.-J.Y.); byronlai@uab.edu (B.L.); deliawang214@gmail.com (F.W.); yumikim@uab.edu (Y.K.); robmotl@uab.edu (R.W.M.); jrimmer@uab.edu (J.H.R.); 3 Department of Physical Therapy, School of Health Professions, University of Alabama at Birmingham, Birmingham, AL 35294, USA; 4 Tanner Foundation for Neurological Diseases, Birmingham, AL 35209, USA; ttracy@alaneuro.com; 5 Department of Occupational Therapy, School of Health Professions, University of Alabama at Birmingham, Birmingham, AL 35294, USA

**Keywords:** static balance, dynamic balance, physical functioning, concurrent validity, convergent validity

## Abstract

**Background:** Recent clinical guidelines for adults with neurological disabilities suggest the need to assess measures of static and dynamic balance using the Berg Balance Scale (BBS) and Dynamic Gait Index (DGI) as core outcome measures. Given that the BBS measures both static and dynamic balance, it was unclear as to whether either of these instruments was superior in terms of its convergent and concurrent validity, and whether there was value in complementing the BBS with the DGI. **Objective**: The objective was to evaluate the concurrent and convergent validity of the BBS and DGI by comparing the performance of these two functional balance tests in people with multiple sclerosis (MS). **Methods**: Baseline cross-sectional data on 75 people with MS were collected for use in this study from 14 physical therapy clinics participating in a large pragmatic cluster-randomized trial. Convergent validity estimates between the DGI and BBS were examined by comparing the partial Spearman correlations of each test to objective lower extremity functional measures (Timed Up and Go (TUG), Six-Minute Walk Test (6MWT), Timed 25-Foot Walk (T25FW) test) and the self-reported outcomes of physical functioning and general health using the 36-Item Short Form Health Survey (SF-36). Concurrent validity was assessed by applying logistic regression with gait disability as the binary outcome (Patient Determined Disease Steps (PDDS) as the criterion measure). The predictive ability of two models, a reduced/parsimonious model including the BBS only and a second model including both the BBS and DGI, were compared using the adjusted coefficient of determinations. **Results**: Both the DGI and BBS were strongly correlated with lower extremity measures overall as well as across the two PDSS strata with correlations. In PDDS ≤ 2, the difference in the convergence of BBS with TUG and DGI with TUG was −0.123 (95% CI: −0.280, −0.012). While this finding was statistically significant at a type 1 error rate of 0.05, it was not significant (Hommel’s adjusted *p*-value = 0.465) after accounting for multiple testing corrections to control for the family-wise error rate. The BBS–SF-36 physical functioning correlation was at least moderate and significant overall and across both PDDS strata. However, the DGI–physical functioning score did not have a statistically significant correlation within PDDS ≤ 2. None of the differences in convergent and concurrent validity between the BBS and DGI were significant. The additional variation in 6MWT explained by the DGI when added to a model with the BBS was 7.78% (95% CI: 0.6%, 15%). **Conclusions**: These exploratory analyses on data collected in pragmatic real-world settings suggest that neither of these measures of balance is profoundly superior to the other in terms of its concurrent and convergent validity. The DGI may not have any utility for people with PDDS ≤ 2, especially if the focus is on mobility, but may be useful if the goal is to provide insight on lower extremity endurance. Further research leveraging longitudinal data from pragmatic trials and quasi-experimental designs may provide more information about the clinical usefulness of the DGI in terms of its predictive validity when compared to the BBS.

## 1. Introduction

Multiple sclerosis (MS) is a chronic neurologic disease that affects 1.1 million adults in the United States [1]. This immune-mediated disease can cause demyelination and axonal loss within the central nervous system [2]. As a result, people with MS commonly experience balance and associated mobility dysfunction [3,4] based on muscular weakness, ataxia, and visual or vestibular disturbances [5] that result in a high risk of falls [6].

Walking function and balance ability are linked with the risk of falls and the degenerative course of the disease [7,8,9,10,11], and integrative balance assessments are necessary for informing rehabilitation programs among people with MS [5]. Recently published clinical practice guidelines recommend the use of both static and dynamic balance tests as part of the core outcome measures that should be measured for adults with neurologic conditions undergoing rehabilitation [12]. One of the most common clinical measures of static and dynamic balance is the Berg Balance Scale (BBS) [13]. The BBS involves 14 items that assess a person’s ability to balance while performing activities ranging from sitting unsupported to stool stepping. The BBS has demonstrated strong inter-rater and test-retest reliability in MS (ICC > 0.95) and has strong level I evidence that supports its use for assessing changes in static and dynamic sitting and standing balance [12]. 

The Dynamic Gait Index (DGI) is a less commonly applied measure of balance, but there is evidence that it represents a valid tool for assessing dynamic balance during walking in people with MS [14,15,16] and has demonstrated excellent test-retest [17], inter-rater (ICC = 0.98), and intra-rater reliability (ICC = 0.76–0.98) [16]. The strength of the DGI is that it is based on a person–environment model of mobility disability (i.e., where mobility disability is determined by both the individual and environment) [18]. To that end, the DGI contains seven items focused solely on an individual’s modification of gait within the context of environmental demands. This makes the DGI an appealing tool for predicting physical function, and it can be used to complement instruments that contain only static balance measures.

There are both clinical and health services implications in the choice of assessments done in clinics. The choice of the instrument is critical given that the outcome measures evaluated are based on time constraints, the complexity of the participant’s diagnosis, and reimbursement guidelines. Both the BBS and DGI are capable of discriminating between individuals with MS who are fallers and non-fallers, and individuals who use and do not use an assistive device [19]. However, the DGI measures only dynamic balance, whereas the BBS measures both static and dynamic balance. Furthermore, the BBS has a strong correlation with the DGI (*r* = 0.78) [19]. While the DGI contains fewer items, it requires stairs and more space to administer when compared with the BBS since the focus is on an individual’s modification of gait within the environment. In light of the similarities and differences between the BBS and DGI, further exploration of the psychometric properties of these outcomes may help therapists determine whether either of these tests are more clinically useful or if there is value in using both tests as a complementary means of balance assessment. 

In this paper, the clinical utility of these two balance measures was defined in terms of the convergent and concurrent validity of scores in pragmatic settings, namely outpatient physical therapy clinics. The paper addressed two research questions: (1) Does the BBS have superior construct and concurrent validity compared to the DGI, or vice-versa? (2) Does including the DGI as a complementary measure to the BBS improve the predictive and discriminative ability of balance assessments compared with the use of the BBS only? We addressed these questions by comparing the correlation and predictive ability estimates of the BBS and DGI with objective measures of lower extremity function as well as self-reported measures of physical function.

## 2. Materials and Methods

### 2.1. Data Source

This ancillary study included baseline-testing data from the first 25% of participants with MS who were recruited for a pragmatic therapeutic exercise clinical trial [20]. The trial included 38 physical therapy clinics spread across different regions in Mississippi, Alabama, and Tennessee. Participants were eligible based on the following criteria: (1) a self-reported diagnosis of MS; (2) a Patient Determined Disease Steps (PDDS) score between 0 and 7 (the PDDS is an ordinal scale ranging from 0 (Normal) to 8 (Bedridden) that measures self-reported disability status [21,22,23] and has a strong correlation of 0.8 with the Expanded Disability Status Scale (EDSS) [21]); (3) ability to use both arms and legs for exercising while standing or seated (inclusive of people with hemiparesis); and (4) physician permission to participate in the study. Exclusion criteria were: (1) visual acuity that precluded exercise with a computer tablet; (2) cardiovascular disease event within the past six months; (3) severe pulmonary disease; (4) renal failure; (5) an active pressure ulcer; (6) currently pregnant; (7) received rehabilitation within the past 30 days; and (8) classified as physically active based on the health contribution score of ≥24 (calculation of only the moderate and vigorous scores) on the Godin Leisure-Time Exercise Questionnaire [24,25]. Data on the DGI were collected at only 14 clinics due to the availability of standardized stairs to administer and measure the DGI. The sample size of this ancillary exploratory study was based on the assumption that each of the 14 clinics would be able to recruit 25% of the total sample size of 820 (assuming equal recruitment across clinics), which was rounded to 76. This would give us 80% power (with a sample size of at least 75) at a hypothesis testing type one error rate of 0.05 to detect an effect size (Cohen’s *d*) of 0.33 when comparing the differences between the respective dependent partial correlations of the BBS and DGI. Furthermore, a sample size above 70 would provide robust estimates of effect size that would help us plan a well-powered probative study in the future [26]. Therapists at each clinic site were trained by research personnel on procedures to administer outcome measures, along with written instructions and checklists to ensure testing consistency. A University Institutional Review Board approved the study and its procedures.

### 2.2. Measures and Procedures

Participants who provided informed consent were asked to complete a demographics and health history questionnaire that included age, sex, race, body height, body weight, and the PDDS scale [21,27]. In addition, participants were instructed to complete the 36-Item Short Form Health Survey (SF-36) as well as tests that assessed lower extremity function and balance. The tests were administered in the following order: (1) Timed Up and Go (TUG); (2) Timed 25-Foot Walk (T25FW); (3) DGI; (4) BBS; and (5) Six-Minute Walk Test (6MWT). All outcome measures were assessed by a trained physical therapist at each clinic site. Each measure was administered with standardized participant instruction scripts and a testing checklist. 

#### 2.2.1. SF-36

The SF-36 [28] is a widely used questionnaire that assesses aspects of health-related quality of life. It contains eight subscales: energy/fatigue, physical functioning, bodily pain, general health perceptions, role limitations due to physical health problems, role limitations due to personal or emotional problems, social functioning, and emotional well-being. Scores range from 0 to 100, with lower scores indicating more disability. We used both the physical functioning subscale score as well as the general health subscale score. 

#### 2.2.2. Timed Up and Go (TUG)

The TUG assesses the time it takes for a participant to rise from a chair, walk to a 3-m mark, turn around, walk back to the chair, and sit down as quickly as possible. The time taken to complete this procedure was recorded in seconds [29]. Each participant was asked to perform three trials and the average time of the three trials was recorded. The test was performed with a chair (height = 45–47 cm), a cone, and a stopwatch. 

#### 2.2.3. Timed 25-Foot Walk (T25FW)

The T25FW measures the time it takes for a participant to walk to a 25-foot mark as quickly as possible. Participants could use assistive devices if needed. Each participant performed two trials, and the average time (in seconds) of the two trials was used. The test is part of the Multiple Sclerosis Functional Composite [30]. Previous studies have reported high inter-rater and test-retest reliability as well as good concurrent validity.

#### 2.2.4. Dynamic Gait Index (DGI)

The DGI is a test of dynamic balance that is administered by a single rater. The rater scores an individual’s performance on eight tasks related to maintaining balance and making modifications in response to external demands while walking [31]. The score is based on a 4-point ordinal scale ranging from 0 (lowest level of function) to 3 (highest level of function). Accordingly, the test items include activities such as walking with head turns, alterations in speed, obstacles, stairs, and a pivot turn. The DGI was performed in an unobstructed hallway or area with a marked 20-foot pathway, a shoebox, two cones of the same height, and stairs (six inches in height with handrails) with a platform at the top to turn around on as well as hand rails. Previous research has reported a minimal detectable change for people with MS of 4.10–5.54 points [19].

#### 2.2.5. Berg Balance Scale (BBS)

The BBS is administered by a single rater who scores an individual’s balance performance from a value of 0 (cannot perform) to 4 (normal performance) on a total of 14 tasks [32]. Tasks incorporate several activities related to static balance such as sitting to standing, retrieving an object from the floor, standing with eyes closed, and standing on one foot. The test was administered with a ruler, two chairs (height: 45–47 cm; one with armrests and one without), a footstool, a 15-foot walkway, and a stopwatch. Previous research has reported a minimum detectable change of seven points in multiple sclerosis [33].

#### 2.2.6. Six-Minute Walk Test (6MWT)

The 6MWT assesses walking endurance, where the distance completed by participants walking over a total of 6 min was documented in feet. The test was administered with a 50-foot path of an unobstructed hallway marked with two cones.

### 2.3. Statistical Analysis

Analysis of data included descriptive summary statistics characterizing the sample. We estimated the percentage of participants who achieved ceiling scores on the BBS and DGI. Convergent validity was assessed through the Spearman partial correlations between the BBS and DGI with the following lower extremity functional outcomes: TUG, T25FW, 6MWT, as well as self-reported health outcomes on the SF-36 physical functioning and general health subscale scores. Differences in the partial correlations were tested and reported using Meng’s test and Zhou’s confidence intervals (CIs) [34,35,36]. All outcome measures were assessed by the same therapist at each clinic site to minimize variation in the administration of the assessment. Furthermore, to account for clinic-level variation in all outcome measures and correlations between participants from the same site, partial correlations were estimated using a two-step process. In step 1, for every outcome measure (e.g., TUG), we first fitted a mixed model with the site as the random effect and age, sex, race, and body mass index (BMI) as the independent fixed effect with the outcome measure(s) as the dependent variable. From each of the fitted models, we then estimated the residuals corresponding to the respective outcome measures (BBS, DGI, TUG, T25FW, 6MWT, SF-36 physical functioning, and SF-36 general health). Residuals of BBS, DGI, TUG, T25FW, 6MWT, SF-36 physical functioning, and SF-36 general health were used to estimate the partial Spearman correlations between the BBS and DGI with the self-reported health measures and measures of lower extremity function. The correlations were then adjusted for race, gender, clinic, age, and BMI. We further tested whether PDDS was an association modifier by creating two strata based on PDDS (PDDS ≤ 2, and PDDS > 2). Separate regression models similar to step 1 were fitted for both the BBS and DGI, but now with an interaction term between the binary PDDS-strata variable with the BBS and DGI, respectively. The dependent variables were the lower extremity functional outcomes and self-reported physical functioning and general health scores. Since the PDDS was a moderator/association modifier for at least some of the dependent variables, we further estimated the partial correlations for subgroups PDDS ≤ 2 and PDDS > 2. The choice of Spearman correlation was to have estimates of correlations that are robust to the violation of normality assumptions and outliers. Meng’s test and Zou’s CIs for differences in the correlated coefficients were undertaken using the R package cocor [34,36]. Since we performed multiple tests to compare the convergent validities, we also performed correction for multiple testing and calculated Hommel’s adjusted *p*-value to control for the family-wise error rate [37]. We considered all of the 15 hypothesis tests conducted to compare the respective partial correlations between the BSS and DGI as part of the family of hypotheses.

To compare the concurrent validity, we established a criterion of gait disability defined as PDDS > 2 versus PDDS ≤ 2 (no gait-related disability). Two competing non-nested logistic regression models were fitted with one including the BBS only and the other including the DGI only with the set of covariates as defined earlier. We estimated McFadden’s pseudo-*R*^2^ for both of these models and estimated the differences in these pseudo-*R*^2^ along with 95% bootstrap CIs.

Finally, for each of the dependent variables (TUG, 6MWT, T25FW, SF-36 physical functioning, and SF-36 general health), we assessed to what extent the DGI in the presence of the BBS increased the variation explained in the lower extremity functional outcomes and self-reported outcomes. We ran two nested linear regression models with the default-reduced model with the BBS as the only balance measure as an independent variable with age, gender, race, BMI, and the clinics as covariates. The second augmented model included both the DGI and BBS as independent variables in addition to the covariates present in the reduced model. We estimated the differences in the adjusted *R*^2^ between models 2 and 1 and generated 95% bootstrap CIs based on 1000 bootstrap replicates. 

All of the analyses were conducted using SAS 9.4 (SAS Institute, Inc., Cary, NC, USA) or in R, and 95% CIs were estimated.

## 3. Results

The participant demographics are shown in Table 1. At the end of the 25% baseline-testing milestone of the clinical trial, the sample from 14 clinics consisted of 75 participants. The average age was approximately 50 years with an interquartile range of 15. Over 90% of the participants were women and the majority of these participants were Caucasian. Over 77% of these participants had a relapsing-remitting type of MS. The median PDDS in this sample was two with a range of 0 to 7.

**Figure 1:**
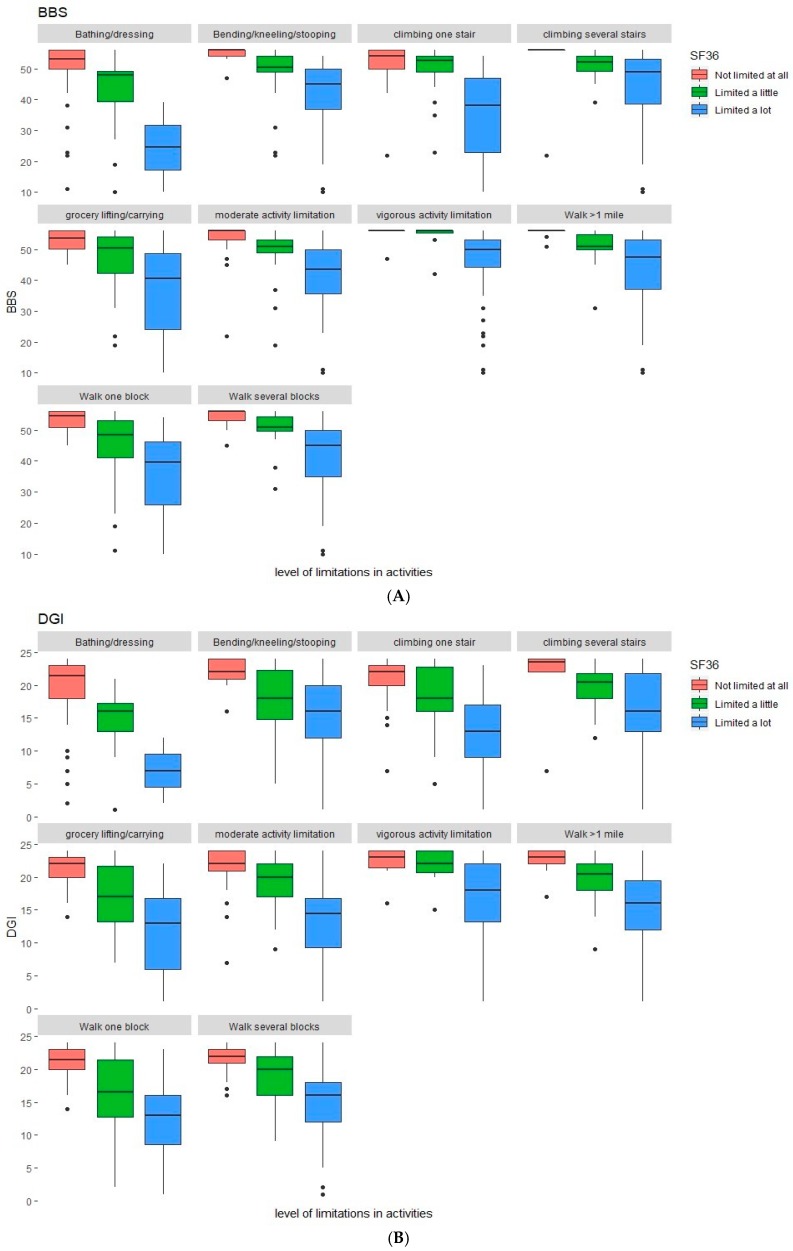
The BBS and DGI relationship items of SF-36 Physical Functioning. Panel (**A**): BBS and SF-36 Physical Functioning Items. Panel (**B**): DGI and SF-36 Physical Functioning Items.

The descriptive statistics of the assessments are shown in Table 2. In contrast to the BBS that had 12.0% ceiling scores, the DGI had 22.7%. The number of participants with a floor effect was two and one for the BBS and DGI, respectively. The median BBS score was 51 with an interquartile range of 10. The median DGI score was 20 with an interquartile range of eight. Figure 1A,B indicate the relationship between the BBS and DGI with specific items of the SF-36 physical functioning measures. 

There was a strong partial correlation of 0.823 (CI: 0.733, 0.885) between the DGI and BBS in the overall sample. The partial Spearman correlations between the DGI and BBS with other functional measures are reported in Table 3. The correlations between the DGI and lower extremity functional outcomes were strong and statistically significant. Among the three lower extremity outcomes of TUG, 6MWT, and T25FW, the DGI–6MWT correlation was strongest with a correlation of 0.763 (CI: 0.645, 0.842). While the DGI–SF-36 physical functioning correlation of 0.570 (CI: 0.390, 0.704) was strong, the DGI–SF-36 general health measure was weak (0.182, CI: −0.048, 0.392) and not statistically significant. The correlations between the BBS and TUG, 6MWT, and T25FW were similar in magnitude to the DGI with the strongest correlation found between BBS and TUG (−0.774, CI: −0.850, −0.660). Similar to the DGI, the BBS–SF-36 physical functioning correlation was strong (0.597, 95% CI: 0.425, 0.724). Like the DGI, the correlation between the BBS and SF-36 general health measure was weak and not statistically significant. None of the differences in the respective correlations between the BBS and DGI were statistically significant at a hypothesis testing error rate of 0.05.

Among those with a PDDS score of ≤2 (i.e., no gait problems), the DGI and BBS had a partial correlation of 0.745 (CI: 0.570, 0.855). In this subgroup, amongst the lower extremity functional measures, correlations of the DGI and BBS with the TUG lower extremity measure were the strongest and statistically significant. The DGI–TUG had a correlation of −0.745 (95% CI: −0.853, −0.565) and the BBS–TUG correlation was −0.868 (95% CI: −0.926, −0.762). Overall, the correlations between the DGI and lower extremity functional outcomes and the BBS and lower extremity functional outcomes were strong and similar in magnitude. The correlation for the DGI with the SF-36 physical functioning was small to moderate and not statistically significant (0.258, 95% CI: −0.052, 0.519), while the corresponding correlation with the BBS was moderate to strong and statistically significant (0.403, 95% CI: 0.109, 0.627). Correlations of the DGI and BBS with SF-36 general health were moderate to none in magnitude and not statistically significant with −0.031 for DGI and 0.012 for BBS. Only the difference between the BBS–TUG and DGI–TUG correlations was statistically significant based on the raw *p*-value (0.031). However, this difference was not statistically significant (Hommel’s adjusted *p*-value = 0.465) after accounting for multiple testing corrections and controlling for the family-wise error rate. Even if we made a less conservative assumption of only five hypothesis tests as part of the family of hypotheses (overall, PDDS ≤ 2, PDDS > 2), the adjusted *p*-value was not statistically significant (*p* = 0.155). From a descriptive standpoint, the BBS–TUG correlation in those with a PDDS ≤ 2 was the strongest and explained 75.3% of the variation in the TUG. 

Among those with a PDDS score >2 (onset of gait disability), the partial correlation between the DGI and BBS was 0.835 (CI: 0.682, 0.913). Both the DGI and BBS had the strongest correlations with the 6MWT (DGI–6MWT: 0.813, CI: 0.645, 0.902; BBS–6MWT: 0.760, CI: 0.557, 0.873). The BBS and DGI correlations with the T25FW and TUG were also strong and similar in magnitude. In this subgroup, the correlations of both the DGI and BBS with the SF-36 physical function score were moderate to strong and statistically significant. However, the correlations with the SF-36 general health score remained null to small with correlations of −0.083 for DGI and −0.205 for BBS. None of the differences in correlations was statistically significant at the 0.05 level. 

Concurrent validity was the estimated adjusted McFadden’s pseudo *R*^2^*s* from two competing non-nested logistic regression models where the dependent variable was defined as gait disability (PDDS > 2) and no gait disability (PDDS ≤ 2). The two competing models had the same covariates with one model having the BBS as the only balance assessment predictor and the second model having both the DGI and BBS as the predictors. The difference in the pseudo *R*^2^ between the BBS and DGI models was −0.026 (95% CI: −0.179, 0.085). The pseudo R^2^ for the BBS was 0.351 (95% CI: 0.189, 0.566) and for the DGI was 0.384 (95% CI: 0.213, 0.618) 

Finally, we assessed the improvement in the ability of a regression model that incorporated both the DGI and BBS scores as independent variables to predict scores for TUG, T25FW, 6MWT, SF-36 physical functioning, and SF-36 general health. This was done by comparing the adjusted *R*^2^ of two nested regression models, one with the BBS only and the other with the DGI and BBS (results shown in Table 4). The increase in the adjusted *R*^2^ of the model that included the DGI in addition to the BBS was very small across all functional measures and self-reported physical functioning and general health measures, except for the 6MWT. We observed a statistically significant increase of 0.078 (95% CI: 0.003, 0.151) in the adjusted R^2^ when predicting the 6MWT scores.

## 4. Discussion

Assessment in clinical settings for patients with MS presents many challenges associated with time, cost, and utility. Balance testing is one of the hallmark measures of clinical assessment for patients with MS, yet there is limited understanding about the clinical utility of various balance measures. The current recommendations suggest that the DGI be included as one of the core outcome measures along with the BBS [12]. In outpatient therapy, therapists providing these services to Medicare beneficiaries are expected to report functional data. However, specific assessment tools are not prescribed and therapists are expected to use professional and clinical judgement when selecting outcomes. Hence, it was unclear if either of these two measures (BBS and DGI) was superior to the other or if both of these measures were needed to obtain an accurate assessment of balance in patients with MS. For this reason, we described the clinical utility of these two balance measures in terms of two research questions: (1) Does the DGI have superior convergent and concurrent validity compared to the BBS, or vice-versa? (2) Does including the DGI as a complementary measure to the BBS improve predictive and discriminative ability of balance assessment compared to use of the BBS alone? To address these questions, we used validated objective lower extremity functional measures (TUG, T25FT, and 6MWT) to address our study questions as well as self-reported SF-36 measures of physical functioning and general health scores and a binary outcome of gait disability using the PDDS criterion of PDDS > 2 as having gait disability and PDDS ≤ 2 as no gait disability. Overall, our findings demonstrated that there was no clear evidence of profound superiority between the BBS and DGI except potentially in PDDS ≤ 2. 

The TUG was considered as the most appropriate comparator for lower extremity functional mobility and was used to assess convergence with the DGI and BBS. Our results indicated that the BBS had a consistently higher correlation with the TUG and T25FW (two measures of lower extremity strength and mobility) when compared to the DGI. The DGI correlation coefficients for the TUG and T25FW were similar to those reported previously in people with MS (DGI correlation coefficients of −0.762 and −0.778, respectively) [15] and (−0.809 and −0.800) [38]. In PDDS > 2, the DGI appeared to have greater convergence with the 6MWT when compared to TUG and T25FW, a finding similar to that reported previously [38]. The BBS–TUG convergence seemed to be greater than the DGI–TUG convergence; the difference was statistically significant when multiple testing was not considered as an issue. However, this finding was not statistically significant when the evidence threshold was more stringent to control for the family-wise error rate. The correlation between the BBS and SF-36 physical functioning score was consistently higher than that of the DGI and SF-36 score. In participants with PDDS ≤ 2 scores (no gait disability), the partial correlation between the BBS and physical functioning score was almost 56% higher than the DGI and SF-36 physical functioning score. In fact, the DGI was not statistically significantly correlated with SF-36 PF while the BBS had a statistically significant correlation in the PDDS ≤ 2. 

Concurrent validity was measured by defining the criterion of gait disability described as gait disability (PDDS > 2) versus no gait disability (described as PDDS ≤ 2). We estimated McFadden’s pseudo-*R*^2^ for two competing models with the BBS and DGI with a binary outcome of gait disability. Descriptively, the DGI had a slightly greater pseudo-*R*^2^ than the BBS, but the difference was very small and not statistically significant. We assessed whether including the DGI as a complementary measure to the BBS improved the ability to predict lower extremity functions and patient reported outcomes when compared to the use of the BBS alone. We found that including the DGI in the model with the BBS improved the predictive ability minimally across most outcomes except the 6MWT. The ability to predict the 6MWT improved when the DGI was used in combination with the BBS. Taken together, the study findings suggest that therapists can use either the BBS or DGI to predict lower extremity function in people with MS, particularly for people with MS who experience gait or mobility disability (PDSS > 2). The DGI appeared to be less useful than the BBS for predicting lower extremity function in people who had mild or no symptoms of gait or mobility disability (PDDS ≤ 2), as indicated by our analyses and the higher observed ceiling effects for the DGI. However, when walking endurance is an outcome of interest, as is often the case within outpatient rehabilitation, our findings suggest that using both the BBS and DGI could be useful. 

One of the key strengths of this study is that the data were collected from pragmatic real-world settings in 14 clinics across three states in the U.S. Identical equipment (stairs) were used to measure the DGI across these clinics, and the same therapist did all of the assessments for a participant. Each of these therapists underwent standardized training before starting the study. Since the participants were from different parts of Alabama, Mississippi, and Tennessee, the sample has more generalizability than traditional single site studies. The key limitation of this study is that it is based on a cross-sectional study design with the intent of conducting exploratory and descriptive analyses. While we did conduct hypothesis tests to compare correlations, the study was not designed and powered to detect small differences in effect sizes, i.e., in this case, small differences in partial correlations and accounting for multiple testing. However, our study had a sufficiently large sample size to provide robust estimates of effect sizes for future studies. 

Our study explored the clinical usefulness of the DGI by comparing its convergent and concurrent validity with the BBS. We did not find compelling evidence to suggest that either of these two measures is substantially superior to the other. These findings, however, do not suggest that both the BBS and DGI tests provide the same level of clinical assessment. Instead, the evidence we provide about the relationships between the BBS and DGI with lower extremity function and self-reported measure of physical function and general health can help practitioners make an informed decision about selecting one or both of these measures as part of their clinical evaluation process. Both the DGI and BBS are not burdensome to administer when the therapist is familiar with the instructions and the grading scale. The DGI may be perceived by therapists to be quicker, with an administration time of approximately less than 10 minutes. Some negatives associated with the DGI are the need for steps (ideally standardized across clinics), a 20-foot unobstructed walking path, and that it can only be administered to ambulatory participants. Furthermore, for the DGI assessments to be comparable, it was necessary that the clinics used the same type of stairs, which may not happen in pragmatic settings. For example, in our convenience sample, we found that only 14 out of the 38 clinics used the same type of stairs. While some of the differences in correlations and explained variation were small, even modest improvements in predictive ability may have clinical utility [39]. Future studies are required to determine clinically useful differences in the predictive ability of clinical balance assessments. Our study also intends to replicate and validate our findings in the future using data from new participants that will be enrolled in this ongoing trial. This will be especially important to rigorously evaluate and confirm at a stringent threshold of evidence whether the BBS–TUG convergence is significantly better when compared to that of the DGI–TUG. We will also assess predictive validity using the additional longitudinal data. Finally, there may be other utilities of these measures, such as for falls prediction, that have not been studied and compared in this study. 

## 5. Conclusions

These exploratory analyses on data collected in pragmatic real-world settings suggest that neither the BBS or DGI is profoundly superior to the other in terms of its concurrent and convergent validity. The DGI may not have any utility for people with PDDS ≤ 2, especially if the focus is on mobility, but may be useful if the goal is to provide insight on lower extremity endurance. Further research leveraging longitudinal data from pragmatic trials and quasi-experimental designs may provide more information about the clinical usefulness of the DGI in terms of its predictive validity when compared to the BBS.

## Figures and Tables

**Table 1:** Demographic and clinical characteristics of the 75 older adults with multiple sclerosis (MS). BMI = body mass index; PDDS = Patient Determined Disease Steps.

Variable	Descriptive Statistic
Age (years)	49.81 (10.27)
BMI (kg/m^2^)	30.94 (7.56)
Sex (*n*, % female)	68, 90.7%
Race (*n*, %)	
White	61, 81.3%
Not White	14, 18.6%
MS Type (*n*, %)	
Progressive	5, 6.7%
Relapsing-Remitting	58, 77.3%
Unknown	9, 12.0%
Not Reported	3, 4%
PDDS (*n*, %)	
0 = normal	15, 20.0%
1 = mild disability	12, 16.0%
2 = moderate disability	15, 20.0%
3 = gait disability	12, 16.0%
4 = early cane	9, 12.0%
5 = late cane	7, 9.3%
6 = bilateral support	4, 5.3%
7 = wheelchair/scooter	1, 1.3%

**Table 2:** Descriptive characteristics of the measures of physical function, physical activity, and balance of the 75 older adults with multiple sclerosis. DGI = Dynamic Gait Index; BBS = Berg Balance Scale; TUG = Timed Up and Go; 6MWT = Six-Minute Walk Test; T25FW = Timed 25-Foot Walk; SF-36 = 36-Item Short Form Health Survey.

Variable	Mean (SD)	Minimum	Median (IQR)	Maximum
DGI	17.92 (5.74)	1.00	20.00 (8.00)	24.00
DGI % Ceiling	22.67% (N = 17, 17/75)			
DGI % Flooring	1.33% (N = 1, 1/75)			
BBS	47.55 (11.34)	10.00	51.00 (10.00)	56.00
BBS % Ceiling	12.00% (N = 9, 9/75)			
BBS % Flooring	2.66% (N = 2, 2/75)			
TUG (s)	12.02 (9.50)	5.4	9.20 (4.90)	140
6MWT (m)	322.16 (125.37)	13.41	317.60 (165.20)	641.30
T25FW (s)	7.37 (5.01)	3.6	5.95 (3.35)	150
SF-36 Physical functioning	48.69 (20.95)	0.00	45.00 (45.00)	100.00
SF-36 General health	45.20 (22.64)	10.00	40.00 (30.00)	100.00

**Table 3:** Partial correlations with 95% confidence intervals (Spearman partial correlation). Covariates include age, sex, race, BMI, and clinic.

Overall				
Measures	DGI	BBS	Meng’s Test *p*-Value for DGI—BBS Raw *p*-Value, Hommel’s Adjusted *p*-Value	Zhou’s 95% CI for DGI—BBS
TUG	−0.703	−0.774	0.109, 0.791	−0.175, 0.0161
	(−0.800, −0.563)	(−0.850, −0.660)		
6MWT	0.763	0.709	0.226, 0.791	−0.155, 0.035
	(0.645, 0.842)	(0.571, 0.805)		
T25FW	−0.708	−0.755	0.296, 0.791	−0.148, 0.043
	(−0.804, −0.570)	(−0.837, −0.634)		
SF-36 Physical function	0.570	0.597	0.625, 0.791	−0.086, 0.144
	(0.390, 0.704)	(0.425, 0.724)		
SF-36	0.182	0.109	0.290, 0.791	−0.206, 0.061
General health	(−0.0482, 0.392)	(−0.121, 0.327)		
**PDDS ≤ 2**				
* TUG	−0.745	−0.868	0.031, 0.465	−0.280, −0.012
	(−0.853, −0.565)	(−0.926, −0.762)		
6MWT	0.592	0.483	0.241, 0.791	−0.313, 0.075
	(0.345, 0.756)	(0.204, 0.683)		
T25FT	−0.693	−0.773	0.259, 0.791	−0.249, 0.061
	(−0.821, 0.487)	(−0.870, −0.608)		
SF-36 Physical function	0.258	0.403	0.172, 0.791	−0.062, 0.357
	(−0.052, 0.519)	(0.109, 0.627)		
SF-36	−0.015	0.015	0.791, 0.791	−0.186, 0.244
General health	(−0.318, 0.290)	(−0.290, 0.317)		
**PDDS > 2**				
TUG	−0.553	−0.587	0.687, 0.791	−0.227, 0.148
	(−0.750, −0.251)	(−0.770, −0.296)		
6MWT	0.813	0.760	0.377, 0.791	−0.209, 0.073
	(0.645, 0.902)	(0.557, 0.873)		
T25FW	−0.621	−0.651	0.702, 0.791	−0.214, 0.142
	(−0.791, −0.344)	(−0.809, −0.388)		
SF-36 Physical function	0.454	0.490	0.694, 0.791	−0.156, 0.235
	(0.123, 0.686)	(0.169, 0.710)		
SF-36	−0.083	−0.205	0.240, 0.791	−0.317, 0.077
General health	(−0.414, 0.269)	(−0.510, 0.152)		

* While the finding was statistically significant, the adjusted *p*-value based on Hommel’s multiple testing correction was 0.465 assuming these 15 tests as a family of hypotheses. If we made a less conservative assumption of five hypothesis tests as part of the family, the adjusted *p*-value was still not significant (*p* = 0.155).

**Table 4:** Differences in the adjusted *R*^2^ (DGI adjusted *R*^2^–BBS adjusted *R*^2^).

Measures	Adjusted *R*^2^ for Model with BBS Only	Differences in Adjusted *R*^2^
TUG	0.281	0.009 (−0.009, 0.199)
6MWT	0.574	0.078 (0.003, 0.152)
T25FW	0.385	0.004 (−0.008, 0.191)
SF-36 Physical function	0.433	0.007 (−0.008, 0.075)
SF-36 General health	0.153	0.012 (−0.012, 0.108)
